# Middle Ear Administration of a Particulate Chitosan Gel in an *in vivo* Model of Cisplatin Ototoxicity

**DOI:** 10.3389/fncel.2019.00268

**Published:** 2019-06-25

**Authors:** Pernilla Videhult Pierre, Anette Fransson, Marta Alina Kisiel, Peter Damberg, Sahar Nikkhou Aski, Mats Andersson, Lotta Hällgren, Göran Laurell

**Affiliations:** ^1^Division of Audiology, Department of Clinical Science, Intervention and Technology, Karolinska Institutet, Stockholm, Sweden; ^2^Department of Surgical Sciences, Uppsala University, Uppsala, Sweden; ^3^Karolinska Experimental Research and Imaging Center, Karolinska University Hospital, Stockholm, Sweden; ^4^Division of Bioscience and Materials, RISE Research Institutes of Sweden, Södertälje, Sweden

**Keywords:** auditory brainstem response, particulate chitosan, cisplatin, hair cell, hearing loss, intratympanic administration, magnetic resonance imaging, sodium thiosulfate

## Abstract

**Background:**

Middle ear (intratympanic, IT) administration is a promising therapeutic method as it offers the possibility of achieving high inner ear drug concentrations with low systemic levels, thus minimizing the risk of systemic side effects and drug-drug interactions. Premature elimination through the Eustachian tube may be reduced by stabilizing drug solutions with a hydrogel, but this raises the secondary issue of conductive hearing loss.

**Aim:**

This study aimed to investigate the properties of a chitosan-based particulate hydrogel formulation when used as a drug carrier for IT administration in an *in vivo* model of ototoxicity.

**Materials and Methods:**

Two particulate chitosan-based IT delivery systems, Thio-25 and Thio-40, were investigated in albino guinea pigs (*n* = 94). Both contained the hearing protecting drug candidate sodium thiosulfate with different concentrations of chitosan gel particles (25% vs. 40%). The safety of the two systems was explored *in vivo*. The most promising system was then tested in guinea pigs subjected to a single intravenous injection with the anticancer drug cisplatin (8 mg/kg b.w.), which has ototoxic side effects. Hearing status was evaluated with acoustically evoked frequency-specific auditory brainstem response (ABR) and hair cell counting. Finally, *in vivo* magnetic resonance imaging was used to study the distribution and elimination of the chitosan-based system from the middle ear cavity in comparison to a hyaluronan-based system.

**Results:**

Both chitosan-based IT delivery systems caused ABR threshold elevations (*p* < 0.05) that remained after 10 days (*p* < 0.05) without evidence of hair cell loss, although the elevation induced by Thio-25 was significantly lower than for Thio-40 (*p* < 0.05). Thio-25 significantly reduced cisplatin-induced ABR threshold elevations (*p* < 0.05) and outer hair cell loss (*p* < 0.05). IT injection of the chitosan- and hyaluronan-based systems filled up most of the middle ear space. There were no significant differences between the systems in terms of distribution and elimination.

**Conclusion:**

Particulate chitosan is a promising drug carrier for IT administration. Future studies should assess whether the physical properties of this technique allow for a smaller injection volume that would reduce conductive hearing loss.

## Introduction

Cisplatin is a first-generation, platinum-based chemotherapeutic agent widely used in solid tumor treatment. However, cisplatin may cause permanent hearing loss and tinnitus, limiting its therapeutic use. There is no established pharmacological method to prevent or treat cisplatin-induced ototoxicity.

At the cellular level, cisplatin-induced ototoxic effects are often manifested as loss of the cochlear outer hair cells (OHCs), as shown in experimental animals (Rybak et al., 2007). Excessive levels of reactive oxygen species play a key role in cisplatin-induced ototoxicity (Sheth et al., 2017), while systemic co-administration of cisplatin with antioxidants can reduce ototoxicity in experimental animals (Campbell et al., 1996; Dickey et al., 2004; Fransson et al., 2017). However, developing a safe antioxidant treatment to protect the hearing of cancer patients subjected to cisplatin therapy is more complicated. Antioxidant treatment can reduce cisplatin’s antineoplastic efficacy since antioxidants may chemically interact with cisplatin (Videhult et al., 2006) and/or promote tumor growth (Sayin et al., 2014) and/or tumor metastasis (Le Gal et al., 2015). Another issue is that at least two barrier systems, the blood-perilymph barrier and intrastrial fluid-blood barrier, limit transportation of otoprotectors from systemic circulation to the cochlear compartments (Cohen-Salmon et al., 2007; Shi, 2016). Both of these problems may be circumvented by middle ear (intratympanic, IT) drug administration.

The round and oval windows are the main routes of drug transport from the tympanic cavity to the inner ear (Salt et al., 2016). Some drugs are likely also absorbed by the middle ear mucosa, which may provide an alternative transportation route to the inner ear. One problem with IT drug delivery is that a large portion of the solution may flow through the Eustachian tube and be swallowed. There are several potential methods to increase the amount of drug reaching the cochlea. However, repeated IT application may be limited by round window edema (Saber et al., 2009) and is associated with increased risks of middle ear infection and chronic perforation of the tympanic membrane. Direct administration of a drug onto the round window membrane involves unnecessary surgical trauma and may have deleterious effects on the inner ear. In the present study, the risk of premature elimination from the tympanic cavity was reduced by using a hydrogel as a drug carrier.

IT administration induces temporary conductive hearing loss, a factor which must be accepted by patients. Furthermore, ideally, a drug carrier should degrade in a controlled manner without inducing any side effects in the middle and inner ears. Here, we assessed the protective effects of a chitosan-based delivery system for sodium thiosulfate to inhibit cisplatin-induced ototoxicity over a 10-day period. The carrier was a homogenously deacetylated chitosan with physical and immunological properties different to those of traditional, heterogeneously deacetylated chitosans. The specific aim of the present study was to investigate a chitosan formulation, based on a dispersed particulate hydrogel in an aqueous suspension, as a drug carrier for IT administration in an *in vivo* model of ototoxicity.

## Materials and Methods

### Study Design

The study consisted of three parts. The first part investigated the safety of a chitosan-based delivery system for IT administration by exploring its effects on auditory brainstem responses (ABRs) to air-conducted pure tones and on hair cells. Two different candidate gel formulations were used, herein referred to as Thio-25 and Thio-40. These formulations differed in terms of the concentration of chitosan particles: 25% in Thio-25 and 40% in Thio-40. Guinea pigs were randomly assigned to one of two treatment groups: Thio-25 (*n* = 15) or Thio-40 (*n* = 15). Both groups received a single unilateral IT injection of sodium thiosulfate (100 mM) in a chitosan-based hydrogel formulation. The contralateral ear served as a control (no IT injection). ABR was measured prior to and 7 and 10 days after IT injection. Neither formulation caused hair cell loss, and ABR threshold shifts in the Thio-25 group were milder than in the Thio-40 group. Thio-25 was therefore used in the second part of the study.

The second part investigated the protective effects of Thio-25 IT administration on cisplatin-induced ototoxicity. Guinea pigs were randomly assigned to three treatment groups: Thio-25-cispt (*n* = 15), Placebo-25-cispt (*n* = 15), or NaCl-cispt (*n* = 15). All three groups received a single intravenous (i.v.) injection of cisplatin [8 mg/kg body weight (b.w.)]. One hour earlier, the Thio-25-cispt group was given a single unilateral IT injection of Thio-25, while the Placebo-25-cispt group received the vehicle only (Placebo-25), i.e., the same gel formulation (25% chitosan gel particles) but without sodium thiosulfate. The NaCl-cispt group was given an IT injection of sodium chloride (9 mg/mL; NaCl). The contralateral ears were not injected in any group. Auditory function was evaluated by measuring ABR prior to injection and then 10 days later.

Animals were euthanized after the final ABR measurement in both study parts. The cochleae of animals in the Thio-25, Thio-40, and Thio-25-cispt groups were collected for histological analyses.

The third part investigated the behavior of a chitosan-based formulation in the tympanic cavity compared with a hyaluronan-based formulation. Guinea pigs (*n* = 19) received single IT injections of a paramagnetic gel based on chitosan (Chito-Dota) and hyaluronan (Hya-Dota). All animals received Chito-Dota in one ear and Hya-Dota in the contralateral ear. The gels were then visualized *in vivo* using magnetic resonance imaging (MRI), which was carried out on three occasions per animal over a 2-week period after gel injection. The animals were euthanized immediately after the final MRI session.

### Animals

Our experiments involved 94 guinea pigs (Duncan-Hartley, Lidköpings Kaninfarm, Lidköping, Sweden). Both sexes were utilized, and b.w. ranged from 263 to 413 g. Animals were maintained in an enriched environment in small groups on a 12-h light/12-h dark cycle at a temperature of 21°C and 60% humidity. They were given access to water and standard chow *ad libitum* and were allowed to acclimatize for a minimum of 10 days prior to the first experimental procedure. At baseline, all guinea pigs had normal tympanic membranes and hearing as determined by otoscopic examination and ABR assessment. During the experimental procedures, the animals were placed on a homeothermic pad. Animals subjected to cisplatin administration were weighed and hydrated with NaCl (37°C) subcutaneously (s.c., 5 mL) each day after cisplatin exposure. All animal procedures were performed under anesthesia and aseptic conditions and were in accordance with the Swedish national regulations for animal care and use. The experimental protocol was approved by the Regional Ethical Review Board in Uppsala (No. C5/15) and the Regional Ethical Review Board in Stockholm (No. 138/15).

### Anesthesia

In parts one and two of the study, general anesthesia was produced with ketamine [intramuscular (i.m.), 40 mg/kg b.w.; Ketalar, 50 mg/mL; Pfizer AB, Sollentuna, Sweden] and xylazine (i.m.; 10 mg/kg b.w.; Rompun, 20 mg/mL; Bayer Health Care AG, Copenhagen, Denmark). Anesthesia depth was assessed by measuring the pedal reflex, and additional doses of ketamine (25 mg/kg b.w.) were given if needed. Bupivacaine (s.c.; Marcain, 2.5 mg/mL; AstraZeneca, Södertälje, Sweden) was used for local anesthesia. Buprenorphine (s.c.; 0.06 mg; Temgesic, 0.3 mg/mL; Schering-Plow, Kenilworth, NJ, United States) was used as a post-treatment analgesia in animals subjected to cisplatin administration.

In part three of the study, general anesthesia was induced in an induction box using 4–5% isoflurane in a 3:7 oxygen:air gas mixture after pretreatment with atropine (s.c.; 0.02–0.05 mg/kg b.w.; Atropine Mylan, 0.5 mg/mL; Mylan, Stockholm, Sweden) and 100% oxygen for 30 min to reduce respiratory tract secretions. The animal was then quickly transferred to an MRI-compatible rig where anesthesia was maintained using 2–3% isoflurane. Lidocaine (s.c.; Lidocaine Accord, 10 mg/mL; Accord Healthcare AB, Solna, Sweden) was used for local anesthesia. Anesthesia depth was monitored by measuring the pedal reflex and visual inspection of the respiratory rate. Under MRI, anesthesia depth was monitored by automatic assessment of heart and breathing rates.

### Gel Preparation

#### Thio-25, Thio-40, and Placebo-25

To prepare Thio-25, chitosan (4.4 g, degree of *N*-deacetylation 49%, viscosity 365 mPas; Viscosan^TM^, Flexichem AB, Uttran, Sweden) was suspended in 185 mL of distilled water (Baxter Medical AB, Kista, Sweden) and dissolved in 2 M aqueous HCl, which was added dropwise. The pH of the solution was adjusted to neutral with 1 M aqueous NaOH. The total volume was then adjusted to 200 mL with distilled water. The solution was filtered through a 5-μm syringe filter and heat-sterilized at 121°C for 20 min. Then, 12 g of this solution was mixed with a solution of sodium thiosulfate (4.8 g, 100 mM; Acros Organics, Thermo Fisher Scientific, Göteborg, Sweden) and another 7.2 g of distilled water. The resultant solution was then filtered through a 5 μm syringe filter. 3,4-Diethoxy-3-cyclobutene-1,2-dione [Aldrich Merck KGaA, Darmstadt, Germany; 41 μL of a 10% (v/v) solution in ethanol] was then added to 22.8 mL of the chitosan solution, and the mixture was stirred for 15 min at room temperature. The solution was then placed in a heating cabinet at 40°C for 14 days, after which the solidified gel was mechanically processed into 10-μm particles. Then, 1.29 g of gel particles were added to 3.87 g of a 30 mM phosphate buffer containing 100 mM of sodium thiosulfate to yield a 25% (w/w) ViscoGel^TM^ suspension. Thio-40 was prepared using the same method but by mixing 2.08 g particles with 3.12 g of the phosphate/thiosulfate buffer. A placebo gel to Thio-25, referred to as Placebo-25, was prepared in a similar way as Thio-25 but without the addition of sodium thiosulfate.

#### Chito-Dota

Chitosan (609.95 mg, degree of *N*-deacetylation 49%, viscosity 49 mPas; Viscosan^TM^, Flexichem AB, Uttran, Sweden) was suspended in 90 mL of distilled water. Then, 530 μL of 2 M aqueous HCl was added, and the chitosan concentration adjusted to 0.5% with water. DOTA-NHS-ester (117.16 mg; Macrocyclics, Plano, TX, United States) was dissolved in 20.0 mL of distilled water. Then, 14.0 mL of this solution was added to 23.51 mL of the chitosan solution. The pH was adjusted to 7.02 with 1 M NaOH, and the mixture stirred for 2 days. Gadolinium (III) acetate hydrate (216.5 mg; Aldrich, Merck KGaA) was dissolved in distilled water (20.0 mL); 14.6 mL of this solution was then added to the chitosan-DOTA solution, and the mixture was stirred overnight. Then, 270 mg of EDTA (Scharlau, Scharlab, Barcelona, Spain) was added, and the mixture stirred for 30 min. The solution was dialyzed against 3 × 3 L distilled water (Spectra/Por Membrane molecular weight cut-off [MWCO]: 6–8,000). Subsequently, the dialysate was lyophilized; 86.8 mg of the lyophilized powder was dissolved in distilled water, the pH adjusted to 6.7, and the volume adjusted to 6.5 mL. To 3.75 g of this solution, diethoxy-3-cyclobutene-1,2-dione [Acros, Organics, Thermo Fisher Scientific; 14 μL of a 6.5% (v/v) solution in ethanol] was added while stirring. After 15 min of stirring at room temperature, the solution was placed in a heating cabinet at 40°C for 14 days. Finally, the solidified gel was mechanically processed into 10-μm particles, and 2.068 g of gel particles were mixed with 6.2 mL of 30 mM phosphate buffer.

#### Hya-Dota

Ethylene diamine (62.2 mg; Sigma-Aldrich; Merck KGaA) was dissolved in 20 mL of distilled water, pH was adjusted to 6.0 with 2 M HCl, and the volume was adjusted to 25.0 mL. *N*-hydroxy succinimide (23.3 mg; Aldrich, Merck KGaA) was then added under vigorous stirring to 209 mg of sodium hyaluronate (Fluka, Merck KGaA). Then, *N*-dimethylaminopropyl-*N* ethyl carbodiimide hydrochloride (10.7 mg; Sigma, Merck KGaA) was added under continuous stirring. This solution was slowly added to the ethylene diamine solution under vigorous stirring, and the reaction mixture was left at room temperature for 4 h. The solution was then dialyzed against 4 × 5 L distilled water (Spectra/Por Membrane MWCO: 2,000), after which the dialysate was lyophilized. Then, 138 mg of the lyophilized powder was dissolved in distilled water; 34.1 mg of DOTA-NHS-ester was added, the pH was adjusted to 6.9, and the mixture was stirred overnight. Then, 41 mg of gadolinium (III) acetate hydrate in 4 mL distilled water was added, and the mixture was stirred overnight. Finally, 270 mg of EDTA was added, and the mixture was stirred for 30 min followed by dialysis against 3 × 4 L of distilled water (Spectra/Por Membrane MWCO: 2,000). The dialysate was subsequently lyophilized, and 54.4 mg of the lyophilized powder was dissolved in 5.44 mL of 30 mM phosphate buffer.

### IT Administration

All animals were subjected to a single gel injection (approximate volume: 0.15 mL) into the auditory bulla following a small paracentesis of the tympanic membrane.

### Cisplatin Administration

Cisplatin (8 mg/kg b.w.; Platinol 1 mg/mL; Bristol-Myers Squibb AB, Solna, Sweden) was injected at a rate of 0.2 mL/min through a catheter (PE50, inner diameter 0.58 mm, outer diameter = 0.965 mm; Intramedic, Becton Dickinson, Franklin Lakes, NJ, United States) inserted into the right jugular vein toward the heart. To rinse the catheter, 1 mL of sterile saline was administered immediately after cisplatin injection. The catheter was subsequently removed, the jugular vein was ligated, and the skin was sutured.

### Hearing Thresholds

Auditory function was quantified by determining the hearing thresholds at 12.5, 20.0, and 30.0 kHz with air-conducted acoustically evoked ABR. Each animal was placed in a soundproof box. The frequency-specific stimulus signal was generated through a signal analyzer (Tucker-Davis Technologies, Alachua, FL, United States) controlled by a computer and presented through an electrostatic speaker (EC1, Tucker-Davis Technologies). The speaker was connected to a 10-cm tube positioned in the ear canal of each guinea pig. Neural responses were then collected using three subdermal electrodes: one placed at the vertex (active), one on the mastoid (reference), and a ground electrode on the lower back. The ABR threshold was defined as the lowest stimulus intensity that produced a reproducible response for ABR wave II, which was visualized at the same latency after an average of 1,000 recordings.

### Morphology

After the final ABR assessment, animals were deeply anesthetized with sodium pentobarbital [25 mg/kg, intraperitoneal (i.p.); Allfatal vet., 100 mg/mL; Omnidea AB, Stockholm, Sweden] and subsequently decapitated. In the Thio-25, Thio-40, and Thio-25-cispt groups, the temporal bones were removed and the bulla opened to expose the cochleae. Small fenestrations were made in the apex and round window, and 4% phosphate-buffered formaldehyde was gently flushed through the cochlea. Surface preparation was performed as previously described (Fransson et al., 2017). After analyzing all inner hair cells (IHCs), OHCs, and scar formations, the proportion (%) of hair cell loss per millimeter distance from the round window was calculated and plotted in cytocochleograms.

### MRI

As described in section “Study Design”, guinea pigs (*n* = 19) received a single IT injection of Chito-Dota in one ear and Hya-Dota in the contralateral ear. Each animal underwent MRI immediately after injection and on two more occasions over 2 weeks. Anesthetized animals were placed in the supine position with the middle ear cavity situated within the sensitive region of a four-channel phased array coil originally designed for rat heart imaging (Rapid Biomedical, Würzburg, Germany) within an MR-compatible animal holder. The bed was positioned in the isocenter of a horizontal 9.4 T Agilent magnet equipped with a 12-cm inner diameter gradient system with a maximum gradient strength of 6000 mT/m and an actively tuned birdcage coil (RAPID Biomedical, Würzburg, Germany). Respiration rate was monitored throughout the experiment, and core body temperature was maintained at 37°C using feedback-controlled warm air (SA Instruments, Stony Brook, NY, United States). T1-weighted 3D images were acquired with a gradient echo 3D (GE3D) sequence: repetition time, 5.16 ms; echo time, 2.60 ms; number of averages, 4; flip angle, 20°; data matrix, 256 × 256 × 348; field of view (FOV), 38.4 × 38.4 × 57.6 mm^3^; resolution scan duration, 33 min and 51 s including two dummy scans.

The bias field was characterized from the ratio of blurred (1.6% of FOV) 3D images acquired separately with the surface and volume coils using identical parameters as for the high-resolution images, apart from a reduced matrix size of 256 × 192 × 192.

After imaging, the animals were sacrificed by an i.p. injection of sodium pentobarbital, followed immediately by decapitation.

All MRI images were bias-field corrected using low-resolution and blurred volume and surface coil images. Intensities and volumes were estimated by segmenting areas in the image where there was a contrast difference using ImageJ (version 1.43; National Institutes of Health, Bethesda, MD, United States) and ITK-snap [version 3.4^1^ (Yushkevich et al., 2006)].

### Statistics

To investigate effects on hearing thresholds, mixed linear modeling was used to account for heteroscedasticity and autocorrelation in terms of time point, frequency, and IT administration (when both ears of an animal were included in the analysis). Frequency was included as a continuous variable. The model with the lowest maximum likelihood and fewest estimated parameters was considered to have the best fit; this was determined with a likelihood ratio chi-square test.

Repeated measures analysis of variance (ANOVA) was used to analyze the percentage loss of OHCs in each row (three levels: rows 1, 2, and 3) as the within-subjects effect and the two types of IT administration (two levels: Thio-25 and none) as the within-subjects effect.

An alpha level of 0.05 was used throughout, and all tests were two-sided. All calculations were performed in IBM SPSS Statistics (v. 23, release 23.0.0.0, 64-bit edition for Mac; Armonk, NY, United States).

## Results

### Study Part One

#### Hearing Thresholds

**FIGURE 1:**
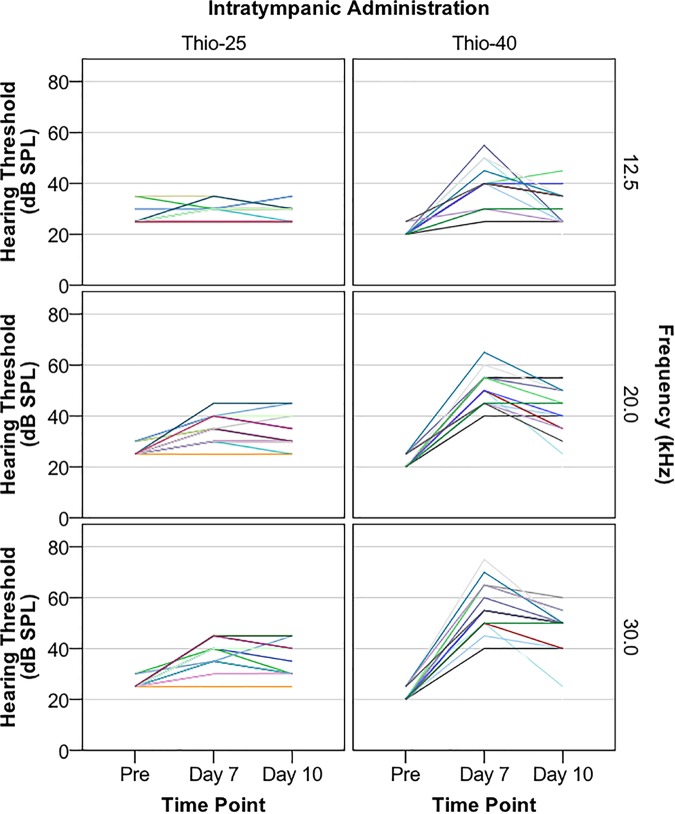
Guinea pigs were subjected to a single, unilateral intratympanic (IT) injection of sodium thiosulfate (100 mM) in a particulate chitosan gel with a gel particle concentration of 25% (Thio-25 group, *n* = 15) or 40% (Thio-40 group, *n* = 15). Electrophysiological hearing thresholds assessed with air-conducted acoustically evoked auditory brainstem response (ABR) were measured at 12.5, 20.0, and 30.0 kHz before (pre) and 7 and 10 days after IT administration. Hearing threshold (decibel sound pressure level, dB SPL) vs. time point stratified by IT administration and frequency is shown. Each color represents one animal. Fewer colors than 15 per graph are due to overlapping lines.

The first part of the study investigated conductive hearing loss induced by unilateral IT injection of two sodium thiosulfate-containing particulate chitosan gels, Thio-25 and Thio-40. The hearing thresholds of each animal measured before and after injection are presented in Figure 1. Mixed linear modeling was performed using hearing threshold as the outcome variable and IT administration (two levels: Thio-25 and Thio-40) as the effect, and frequency and time point (three levels: pretreatment, day 7, and day 10) as repeated effects. In the final model, time point was also used to model a random slope. There was a significant main effect of frequency (*F* = 146.18, *p* < 0.0001) and significant interactions between frequency and IT administration (*F* = 14.87, *p* < 0.001), frequency and time point (*F* = 69.00, *p* < 0.0001), and IT administration and time point (*F* = 24.85, *p* < 0.0001). The estimates of fixed effects are given in Table 1A, and pairwise comparisons are shown in Tables 1B,C. Both Thio-25 and Thio-40 increased the electrophysiological hearing thresholds, although Thio-25 did this to a significantly lesser extent than Thio-40. The hearing thresholds of both groups were significantly higher on days 7 and 10 compared to pretreatment.

**Table 1A:** Mixed linear modeling of associations of the hearing thresholds presented in Figure 1 with IT administration, frequency, and time point.

Estimates of fixed effects on hearing threshold (dB SPL)						
**Parameter**		***β***	**SE of *β***	**t**	***p*-value**	**95% CI**

Intercept		23	1.9	12.51	<0.0001	20 to 27
Frequency^#^		0.72	0.08	8.59	<0.0001	0.55 to 0.89
***Interactions***						
Time Point and IT	*Pre and Thio-25*	4.6	2.1	2.17	0.03	0.38 to 8.9
	*Pre and Thio-40*	-3.4	1.9	-1.75	NS	-7.23 to 0.48
	*Day 7 and Thio-25*	-2.9	2.8	-1.03	NS	-8.54 to 2.69
	*Day 7 and Thio-40*	8.2	2.5	3.37	0.001	3.4 to 13
	*Day 10 and Thio-25*	-2.0	2.0	-1.01	NS	-6.1 to 2.0
	*Day 10 and Thio-40*	Ref				
Time Point and Frequency^#^	*Pre and Frequency*	-0.65	0.08	-7.73	<0.0001	-0.82 to -0.48
	*Day 7 and Frequency*	0.11	0.11	1.04	NS	-0.11 to 0.33
	*Day 10 and Frequency*	Ref				
IT and Frequency^#^	*Thio-25 and Frequency*	-0.14	0.04	-3.86	<0.001	-0.21 to -0.07
	*Thio-40 and Frequency*	Ref				


*Estimates of fixed effects on the outcome variable, hearing threshold (dB SPL) are shown. Results obtained with mixed linear modeling using electrophysiological hearing threshold based on acoustically evoked ABR as the outcome variable, IT administration as an effect, frequency as a repeated effect, time point as a repeated effect, and time point as an effect for random slope. Covariate type: compound symmetry heterogeneous (fixed effects) and variance components (random effect). *^#^*In kHz. ABR, auditory brainstem response; *β*, coefficient of regression; CI, confidence interval; dB SPL, decibel sound pressure; IT, intratympanic; NS, non-significant; Pre, before IT administration; Ref, reference category.*

**Table 1B:** Pairwise comparisons of the analysis results presented in Table 1A.

Pairwise comparisons of effects for hearing threshold (dB SPL)						
**Time point**	**(I) IT**	**(J) IT**	**Mean difference (I-J)**	**SE**	***p*-value**	**95% CI**

Pre	Thio-25	Thio-40	5.1^∗^	1.0	<0.0001	3.1 to 7.1
Day 7	Thio-25	Thio-40	-14^∗^	1.9	<0.0001	-18 to -10
Day 10	Thio-25	Thio-40	-4.9^∗^	2.0	0.02	-8.9 to -1.0


*CI, confidence interval; dB SPL, decibel sound pressure level; NS, non-significant; pre, before IT administration; SE, standard error of mean difference. ^∗^ signifies *p* < 0.05 (Bonferroni adjusted).*

**Table 1C:** Pairwise comparisons of the analysis results presented in Table 1A.

Pairwise comparisons of effects for hearing threshold (dB SPL)						
**IT**	**(I) Time point**	**(J) Time point**	**Mean difference (I-J)**	**SE**	***p*-value**	**95% CI**

Thio-25	Pre	Day 7	-8.4^∗^	1.3	<0.0001	-12 to -5.1
	Pre	Day 10	-6.9^∗^	1.4	<0.0001	-10 to -3.3
	Day 7	Day 10	1.5	1.4	NS	-1.9 to 4.9
Thio-40	Pre	Day 7	-28^∗^	1.3	<0.0001	-31 to -24
	Pre	Day 10	-17^∗^	1.4	<0.0001	-21 to -13
	Day 7	Day 10	11^∗^	1.4	<0.0001	7.3 to 14


*CI, confidence interval; dB SPL, decibel sound pressure level; NS, non-significant; pre, before IT administration; SE, standard error of mean difference. ^∗^ signifies *p* < 0.05 (Bonferroni adjusted).*

#### Morphology

No significant IHC or OHC loss was found in the first five animals of the Thio-25 (Supplementary Table S1) and Thio-40 (Supplementary Table S2) groups. As the morphological procedure is very laborious, we decided not to count the remaining cochleae. These results indicate that the elevated hearing thresholds presented in section “Hearing Thresholds” were caused by conductive hearing loss.

### Study Part Two

#### Hearing Thresholds

**FIGURE 2:**
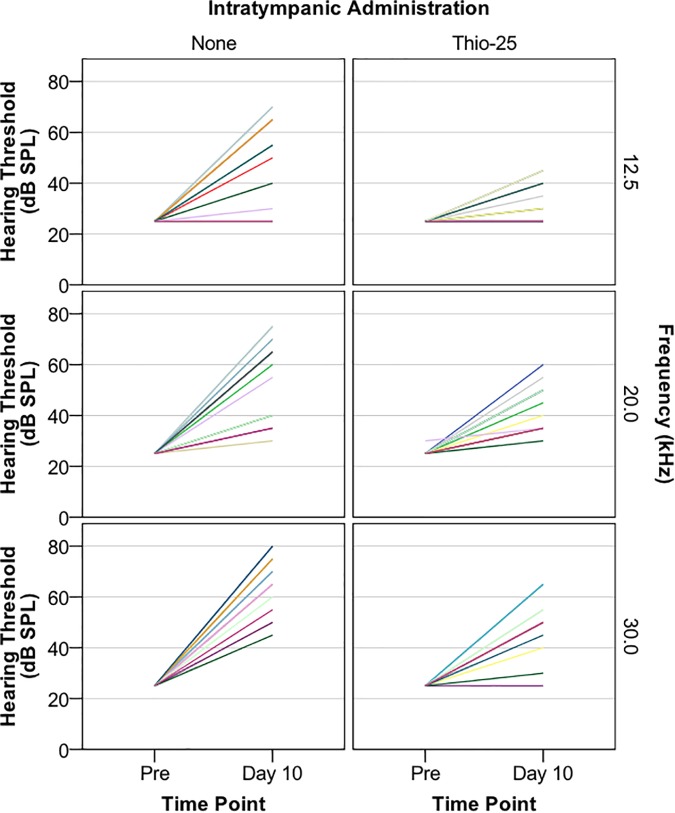
Guinea pigs were subjected to a single, unilateral IT injection of a sodium thiosulfate-containing chitosan-based gel (Thio-25, *n* = 15), while the other ear was left untreated, serving as a control (None). The animals received a single high dose of cisplatin (8 mg/kg b.w., i.v.) 1 h later. Electrophysiological hearing thresholds (in decibel sound pressure level, dB SPL) assessed with air-conducted acoustically evoked auditory brainstem response (ABR) at 12.5, 20, and 30 kHz before and 10 days after IT administration are shown. Each color represents one animal. Fewer colors than 15 per graph are due to overlapping lines.

As the IT administration of Thio-25 induced less conductive hearing loss than Thio-40 and because neither treatment caused hair cell loss, the protective effects of Thio-25 on cisplatin-induced ototoxicity were explored. Electrophysiological hearing thresholds before and 10 days after IT administration of Thio-25 followed by i.v. cisplatin injection are shown in Figure 2. Mixed linear modeling was then carried out using hearing threshold as the outcome variable and IT administration (two levels: Thio-25 and none), frequency, and time point (two levels: pretreatment and day 10) as repeated effects. In the final model, time point was also used to model a random slope. A significant main effect of IT administration (*F* = 56.68, *p* < 0.0001) and frequency (*F* = 126.06, *p* < 0.0001), and significant interactions between time point and IT administration (*F* = 33.76, *p* < 0.0001) and between time point and frequency (*F* = 126.38, *p* < 0.0001) were found. The fixed effects estimates are given in Table 2A, and pairwise comparisons are given in Tables 2B,C. In summary, cisplatin injection induced a significant increase in electrophysiological hearing threshold; however, this effect was significantly lower in ears treated with Thio-25 compared to no IT administration.

**Table 2A:** Mixed linear modeling of associations of the hearing thresholds in the Thio-25-cispt group (*n* = 15) presented in Figure 2 with IT administration, frequency, and time point.

Estimates of fixed effects on hearing thresholds (dB SPL)						
**Parameter**		***β***	**SE OF *β***	**t**	***p*-value**	**95% CI**

Intercept		21	2.0	10.80	<0.0001	17 to 25
IT	*None*	16	1.9	8.26	<0.0001	12 to 19
	*Thio-25*	Ref				
Frequency^#^		0.96	0.06	14.79	<0.0001	0.83 to 1.1
***Interactions***						
Time point and IT	*Pre and None*	-12	3.6	-3.26	<0.005	-19 to -4.40
	*Pre and Thio-25*	4.0	2.6	1.51	NS	-1.3 to 9.2
	*Day 10 and None*	Ref				
	*Day 10 and Thio-25*	Ref				
Time point and frequency	*Pre and Frequency*	-0.96	0.09	-11.24	<0.0001	-1.1 to -0.79
	*Day 10 and Frequency*	Ref				


*Estimates of fixed effects on the outcome variable, hearing threshold (decibel sound pressure level; db SPL), are shown. Results obtained with mixed linear modeling using electrophysiological hearing threshold based on acoustically evoked ABR as the outcome variable, IT administration as a repeated effect, frequency as a repeated effect, time point as a repeated effect, and time point as an effect for random slope. Covariate type: first-order factor analytic (repeated effects) and variance components (random effect). ^#^In kHz; ABR, auditory brainstem response; *β*, coefficient of regression; CI, confidence interval; dB SPL, decibel sound pressure; IT, intratympanic; NS, non-significant; Pre, before IT administration; Ref, reference category; SE, standard error of *β*.*

**Table 2B:** Pairwise comparisons of the analysis results presented in Table 2A.

Pairwise comparisons of effects for hearing threshold (dB SPL)						
**Time point**	**(I) IT**	**(J) IT**	**Mean difference (I-J)**	**SE**	***p*-value**	**95% CI**

Pre	None	Thio-25	-0.1	0.8	NS	-1.7 to 1.5
Day 10	None	Thio-25	16^∗^	1.9	<0.0001	12 to 19


*CI, confidence interval; dB SPL, decibel sound pressure level; NS, non-significant; Pre, before IT administration; SE, standard error of mean difference. ^∗^ signifies *p* < 0.05 (Bonferroni adjusted).*

**Table 2C:** Pairwise comparisons of the analysis results presented in Table 2A.

IT	(I) Time point	(J) Time point	Mean difference (I-J)	SE	*p*-value	95% CI
None	Pre	Day 10	-31.6^∗^	2.7	<0.0001	-37 to -26
Thio-25	Pre	Day 10	-16.0^∗^	1.9	<0.0001	-20 to -12


*CI, confidence interval; NS, non-significant; Pre; before IT administration; SE, standard error of mean difference. ^∗^ signifies *p* < 0.05 (Bonferroni adjusted).*

This experiment was repeated using Placebo-25 (the chitosan-based vehicle without sodium thiosulfate) instead of Thio-25. Ten days after cisplatin administration, animals in the Placebo-25 group had symmetrical, bilateral electrophysiological hearing threshold elevations (Supplementary Figure S1), suggesting that the vehicle itself did not offer any otoprotection.

The experiment was then repeated a third time, using NaCl instead of Thio-25. Ten days after cisplatin administration, the animals had symmetrical, bilateral electrophysiological hearing threshold elevations (Supplementary Figure S2). Thus, IT administration had no effect on hearing thresholds.

#### Morphology

**FIGURE 3:**
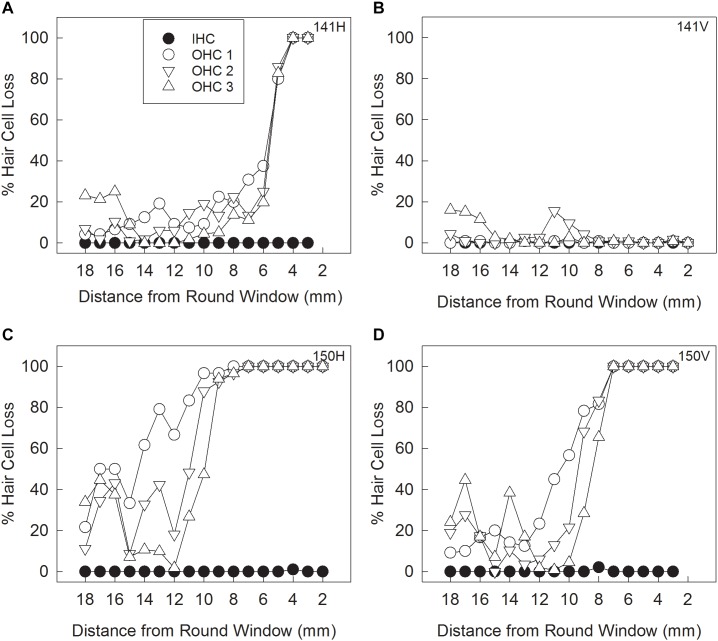
Animals in the Thio-25-cispt group (shown in Figure 2) were euthanized after hearing threshold assessment on day 10. Their cochleae were collected to quantify loss of inner hair cells (IHCs) and outer hair cells (OHCs) in the first (OHC1), second (OHC2), and third (OHC3) rows. Cytocochleogram results from an animal with a loss pattern that was representative of most of the cisplatin-treated guinea pigs subjected to no IT administration **(A)** and to IT administration of Thio-25 **(B)**. Cytocochleogram results from the only cisplatin-treated animal with a large loss in the untreated ear **(C)** and the Thio-25-treated ear **(D)**.

**FIGURE 4:**
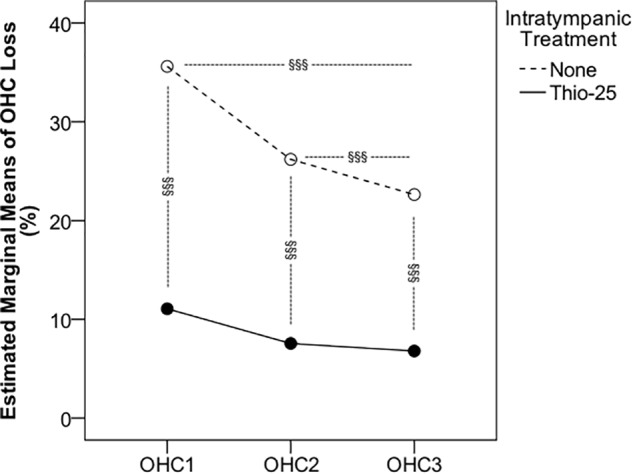
Animals in the Thio-25-cispt group (presented in Figure 2) were euthanized after hearing threshold assessment on day 10. Their cochleae were collected to quantify hair cell loss. Repeated measures ANOVA of the percentage loss of outer hair cells (OHCs) in the first (OHC1), second (OHC2), and third (OHC3) rows are shown for ears injected with a thiosulfate- containing gel (Thio-25) and for the contralateral, non-injected ear (None). ^§§§^ signifies *p* < 0.001.

A representative example of the hair cell loss observed in cisplatin-injected animals that were unilaterally pretreated with IT Thio-25 (group Thio-25-cispt) is shown in Figure 3A,B. One animal showed marked bilateral hair cell loss (Figure 3C,D). The mean percentage losses of OHCs and IHCs in all animals are presented in Table 3. Repeated measures ANOVA revealed a statistically significant difference in terms of OHC loss across rows 1 [*F*(1,14) = 35.868, *p* < 0.001], 2 [*F*(1,14) = 19.368, *p* < 0.001], and 3 [*F*(1,14) = 18.145, *p* < 0.001], as well as statistically significant differences in terms of IT administration [*F*(2,13) = 27.310, *p* < 0.001]. The *post hoc* analysis results are shown in Figure 4. In summary, cisplatin-induced loss of OHCs was significantly less severe in ears subjected to IT Thio-25 compared to no IT administration, which agreed with our electrophysiological hearing threshold results.

**Table 3:** Quantification of hair cell loss in Thio-25-cispt group described in Figure 2.

	Hair cell loss (%)							
	
	OHC1		OHC2		OHC3		IHC	
					
IT	Mean	SD	Mean	SD	Mean	SD	Mean	SD
None	36	24	26	21	23	18	0.0	0.1
Thio-25	11	15	7.6	12	6.8	12	0.8	1.8


*IHC, inner hair cells; IT, intratympanic; OHC1, -2, and -3, outer hair cells by row; SD, standard deviation.*

### Study Part Three

#### Gel Distribution and Clearance

**FIGURE 5:**
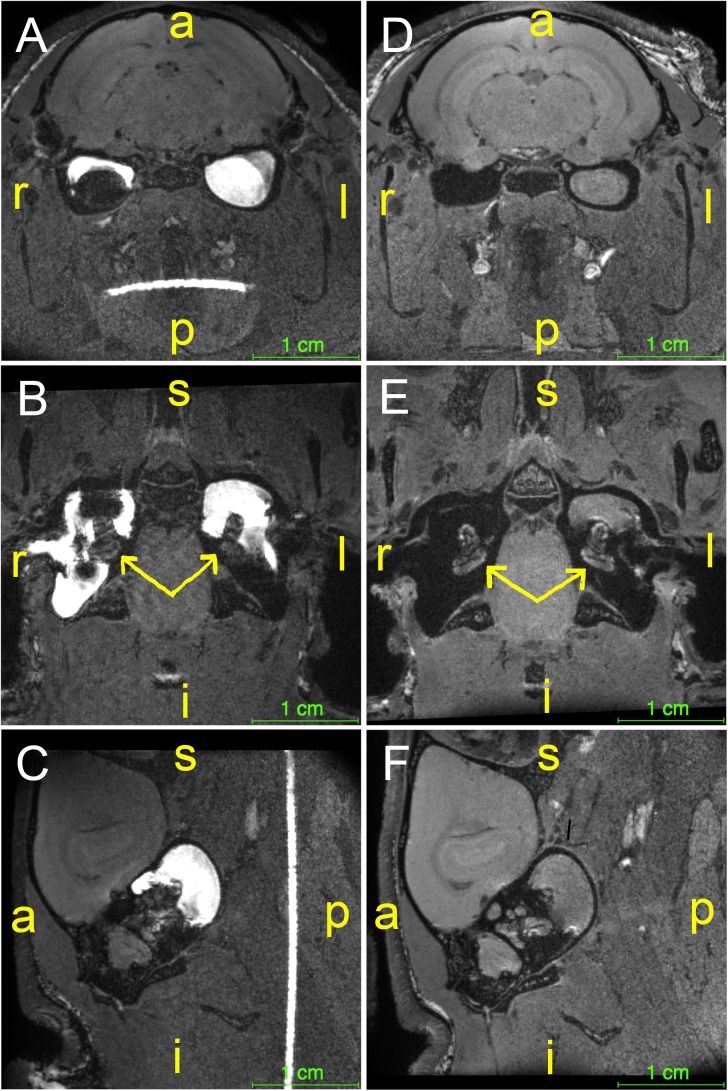
Guinea pigs (*n* = 19) were subjected to IT administration of a paramagnetic chitosan-based gel (Chito-Dota) in one ear and a hyaluronan- based gel (Hya-Dota) in the contralateral ear. The gels were then visualized by magnetic resonance imaging. Different orthogonal viewing planes of 3D data from one animal taken immediately after **(A–C)** and 4 days after **(D–F)** injection of Chito-Dota into the right ear and Hya-Dota into the left ear. The position of each cochlea is indicated with a yellow arrow in **B** and **E**. The sagittal plane in **C** and **F** shows the left ear. Keys (in yellow): a, anterior; i, inferior; l, left; p, posterior; r, right; s, superior.

**FIGURE 6:**
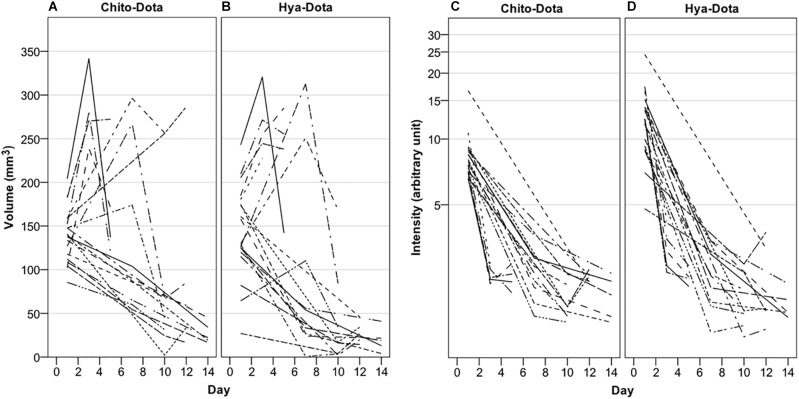
Guinea pigs were subjected to intratympanic IT administration of two different paramagnetic gels, one based on chitosan (Chito-Dota) in one ear and one based on hyaluronan (Hya-Dota) in the contralateral ear. Volume and intensity in the middle ear were explored with magnetic resonance imaging performed on three different occasions in each guinea pig. The volume **(A,B)** and intensity **(C,D)** in the middle ear vs. time curves for each guinea pig are shown. *n* = 19 on day 1, *n* = 5 on day 3, *n* = 4 on day 5, *n* = 9 on day 7, *n* = 8 on day 10, *n* = 5 on day 12, and *n* = 5 on day 14 for both Chito-Dota and Hya-Dota.

In the last part of the study, serial MRI was performed to monitor the middle ear distributions of chitosan and hyaluronan, including clearance from the middle ear cavity over a period of 14 days. The polymers were conjugated with a paramagnetic contrast agent to further improve detection and enable estimation of gel clearance over time. Minutes after injection, the contrast-enhanced gel appeared hyperintense and its location in the middle ear cavity was clearly seen, as seen in Figure 5A–C, which show different orthogonal viewing planes of 3D data from an animal with IT administration of Chito-Dota and Hya-Dota in the right and left middle ears, respectively. At subsequent time points, the intensities were similar to nearby brain tissue, as seen in Figure 5D–F, which were taken 4 days after gel administration and show an empty right middle ear and a mostly fluid-filled left middle ear. Figure 6 shows gel volume vs. time (A and B) and intensity vs. time (C and D) curves of all middle ears. The volume occupied by the gel was larger at the earliest second scanning, i.e., two days after gel administration, compared to the scanning performed immediately after administration of Chito-Dota (Figure 6A) and Hya-Dota (Figure 6B). At later time points, there was a trend for a successive decrease in gel volume. However, there was considerable interindividual variability (represented by the spread shown in Figure 6A,B) at each time point. For example, in 3 of 9 middle ears monitored 6 days after gel injection in each of the Chito-Dota and Hya-Dota groups, the gel volume was larger than immediately after gel injection and thereafter decreased. T1-weighted MR image intensity was dramatically decreased when comparing images acquired at the time of gel administration with those acquired 2 days later (Figure 6C,D). Presumably, the increase in volume and reduction in intensity reflect an initial accumulation of water from the surrounding tissues, thus diluting the contrast agent. Both the distribution and elimination of the chitosan- and hyaluronan-based gels appear to follow similar patterns, and a clear difference between the two forms could not be identified.

## Discussion

IT administration is commonly used in preclinical research. The present large-scale *in vivo* study in guinea pigs provides key data relating to the safety, efficacy, and elimination of an IT drug delivery system composed of an antioxidant incorporated in a particulate hydrogel drug carrier. A sodium thiosulfate-containing, chitosan-based system reduced ototoxic injury induced by a single high dose of cisplatin. Cisplatin is a common anticancer agent that predominantly exerts its effects through DNA-dependent mechanisms (Ghosh, 2019). Evidence from preclinical studies demonstrates that the cisplatin ototoxicity is primarily caused by OHC damage in the cochlea (Laurell and Bagger-Sjöbäck, 1991), and the underlying mechanisms include oxidative stress and mitochondrial dysfunction (Giari et al., 2012; Sheth et al., 2017). Drugs that up-regulate the antioxidant system are therefore considered promising candidates for otoprotection. Two measures were used to explore the ototoxic effects of cisplatin and the efficacy of middle ear administration: assessment of ABR and counting of cochlear hair cells. The primary outcome measure to verify toxicity and assess protective effects was hair cell counting, while the functional capacity of the ear following IT administration was better reflected by ABR assessment. Under normal physiological conditions, the middle ear cavity is filled with gas to optimize sound conduction to the cochlea. When a drug carrier is inserted into the middle ear cavity, air-conducted sound might temporarily be obstructed (conductive hearing loss). Therefore, it is generally desirable to use a technique to estimate bone conduction frequency-specific ABRs as this could provide useful information about middle ear function. However, assessing bone thresholds is difficult in the guinea pig. The first part of the study investigated hair cell loss induced by the drug delivery system. Although ABR assessment showed significantly increased thresholds on days 7 and 10, hair cell counting did not reveal any OHC or IHC loss. This finding corroborates previous results (Saber et al., 2010; Lajud et al., 2015). Taken together, the observed effects of the drug delivery system on hearing thresholds are largely due to conductive hearing loss and agree with a number of earlier *in vivo* studies (Engmér Berglin et al., 2015; Cole et al., 2018). In the second part of the study, the utility of particulate chitosan as a drug carrier for an antioxidant to protect inner ear structures was examined. The choice of sodium thiosulfate was based on positive results from earlier studies (Videhult et al., 2006; Videhult Pierre et al., 2009; Berglin et al., 2011). Sodium thiosulfate (100 mM), in a formulation containing 25% of thiosulfate-containing chitosan gel particles in suspension was used to protect the hearing end organ via a simple injection to the middle ear cavity 1 h before cisplatin administration. As conductive hearing loss did not completely recover within 10 days in the first part of the study, it was evident that hair cell counting represented a more reliable measure of ototoxicity. A partial protective effect on all three OHC rows was observed. Although some OHC loss occurred, it is important to consider that this is the first evidence of otoprotection observed 10 days after a single i.v. administration of cisplatin. On the other hand, short-term *in vivo* studies reported partial protection against cisplatin ototoxicity [see e.g., (Ghosh et al., 2018; Wang et al., 2018)]. Unfortunately, findings from many preclinical studies cannot be translated into clinical trials because of drug toxicity, drug-drug interaction, or the impermeability of the blood-labyrinth barriers. For example, Roldán-Fidalgo et al. (2016) reported that lutein exerted significant otoprotective effects *in vitro* but not *in vivo*. Earlier studies on drug delivery involved short follow-up periods, multiple dosing strategies, and more unreliable methods for administering cisplatin (i.p.). In the second part of the study, the effects of the drug carried itself on cisplatin-induced ototoxicity was also investigated. ABR assessment showed no significant difference between vehicle-treated and untreated ears. Therefore, hair cell loss in vehicle-treated ears was not assessed, largely because the technique is very laborious. As chitosan exhibits antioxidant properties (Ngo and Kim, 2014), it cannot be excluded that chitosan reduced hair cell loss in vehicle-treated ears but the effect was masked by the conductive hearing loss induced by IT administration. In the last part of the study, MRI was used to examine the distribution and elimination of particulate chitosan in comparison to hyaluronan, which is a frequently used vehicle for IT drug delivery [see e.g., (Arriaga and Goldman, 1998; Berglin et al., 2011; Rolland et al., 2019)]. To the best of our knowledge, no previous study has investigated these properties in two gels in the same animal for up to 14 days. Both polymers were conjugated with a gadolinium-containing contrast agent to improve detection. Gel volume changes in the middle ear varied between animals. A typical pattern was increase in gel volume the first days after IT injection, followed by a decrease. At 2 weeks (4 days longer than the protective study in part two), the middle ear was typically clear of fluid, and the MR signal in the inner ear had returned to baseline.

Two major treatment approaches have been employed to circumvent ototoxicity in patients receiving cisplatin-based chemotherapy: systemic and local administration of otoprotector drug candidates. Drugs can be systemically administered during a time window that achieves otoprotection hopefully without compromising antineoplastic efficacy (Brock et al., 2018), or IT injection can deliver otoprotector drugs directly to the inner ear (Marshak et al., 2014; Sarafraz et al., 2018; Rolland et al., 2019). There has been much interest in improving formulations for IT drug delivery. The most appropriate formulation would facilitate drug transport of an otoprotector into the inner ear compartments while minimizing negative effects in the middle ear, including conductive hearing loss. In the present study, the entire middle ear was filled with chitosan. Injecting a smaller volume could be one way to improve chitosan-based delivery systems. Future research should address the effects of injection volume on conduction following IT delivery of a gel. Furthermore, little is known about drug absorption in the middle ear mucosa and how it affects drug transport to the inner ear; future research should address this shortfall. Longitudinal studies for longer than 10 days after cisplatin administration should also be performed to determine whether IT administration can sustain otoprotective effects.

Chitosan is a water-soluble polymer formed by deacetylation of the linear, naturally occurring polymer chitin, which is built up by 1-4-b-linked *N*-acetyl-glucosamine (Ways et al., 2018). Chitosan is biodegradable with low toxicity, and its cationic nature makes it mucoadhesive (Ways et al., 2018). It has been investigated in many types of pharmaceutical formulations (Ways et al., 2018), including drug delivery to the inner ear (Paulson et al., 2008; Lajud et al., 2013). Technically speaking, there are two types chitosans: those with heterogeneous or homogenous acetylation patterns (Sannan et al., 1976). For decades, only heterogeneously deacetylated chitosans were available on the market. They are typically 70–95% deacetylated, which limits their solubility, and precipitate from solution at pH > 6. Heterogeneously deacetylated chitosans are therefore less suitable for IT administration. Although the pH in the middle ear cavity was not measured in the present study, a pH > 6 is expected in the steady-state conditions established in the guinea pig model. Moreover, IT administration of an acidic formulation can exacerbate cisplatin ototoxicity (Tanaka et al., 2003, 2004). The present study use a formulation with homogenously deacetylated chitosan and a pH of 7.4. These chitosans have recently become available and have lower degrees of deacetylation at 35–80%. In addition to the advantageous properties of heterogeneously deacetylated chitosans, new products have additional features including more rapid biodegradation (Shigemasa et al., 1994) and solubility at physiological pH (Vårum et al., 1994). In contrast to the heterogeneously deacetylated chitosans, the homogenously deacetylated chitosans enable the formation of a viscoelastic hydrogel, so called “crushed gels” or “ringing gels.” These gels are slightly cross-linked and form rigid constructs, which can be loaded with drugs, and after solidification they can be mechanically processed into well-separated particles of defined size. ViscoGel^TM^ is an example of such a particulate hydrogel (Franzen et al., 2015) and was clinically evaluated in a vaccine study where it showed a good safety profile (Neimert-Andersson et al., 2014). The present investigation used a chitosan-based, cross-linked sodium thiosulfate-loaded viscoelastic gel suspended in an aqueous thiosulfate-containing buffer. The formulation was designed to combine the most attractive properties of chitosan hydrogels and drug release functionality. We hypothesized that the aqueous phase would enable rapid transport of thiosulfate to the inner ear, while the gel particles would achieve more sustained thiosulfate delivery by increasing its time in the middle ear cavity. The formulations were easily injected into the middle ear cavity and also offer the possibility of fine-tuning drug-release properties by altering gel particle size and/or concentration.

Minimizing cisplatin-induced ototoxicity is of great clinical importance. Many physicians would like to avoid systemic protective treatment as it may interfere with the antineoplastic efficacy of cisplatin (Freyer et al., 2017). A prerequisite for developing otoprotection strategies is knowledge of the inner ear pharmacokinetics of cisplatin. It is well recognized that cisplatin-induced hearing loss is primarily seen in the high-frequency area corresponding to the basal turn of the cochlea. The drug is not uniformly distributed throughout the cochlea; instead, the highest concentration is found in the basal turn (Hellberg et al., 2013), in the vicinity of the round and oval windows. The findings above support the development of local protective treatment via IT administration of one or more otoprotectors. Previous studies suggest that cisplatin is not just transported by passive diffusion; enhanced uptake to inner ear targets involves active transport mechanisms, such as the organic cation 2 and the copper transporter 1 (Ciarimboli et al., 2010; More et al., 2010). IT administration of a drug that could block these transporters in the inner ear might represent an alternative method to reduce cisplatin-induced ototoxicity.

## Conclusion

The results of this preclinical *in vivo* study show that cisplatin ototoxicity can be reduced by the localized administration of the antioxidant sodium thiosulfate in a suspension of a particulate, homogenously deacetylated chitosan. The IT drug delivery system induced conductive hearing loss that was not completely resolved within the 10-days study period, probably due to gel residue remaining in the middle ear. Future research will determine whether the properties of particulate chitosan for drug delivery to the inner ear can be further improved.

## Ethics Statement

The study was carried out in accordance with the recommendations of the Swedish national regulations for animal care and use. The protocol was approved by the Regional Ethical Review Board in Uppsala (No. C5/15) and the Regional Ethical Review Board in Stockholm (No. 138/15).

## Author Contributions

All authors listed have made a substantial, direct and intellectual contribution to the work, and approved it for publication.

## Conflict of Interest Statement

MA is CEO at Flexichem AB, the inventor and current holder of the Viscosan manufacturing process. The remaining authors declare that the research was conducted in the absence of any commercial or financial relationships that could be construed as a potential conflict of interest. The reviewer SH declared a shared affiliation, though no other collaboration, with one of the authors PVP to the handling Editor.

## Footnotes

1 www.itksnap.org
