# Supplementary material for: Middle Ear Administration of a Particulate Chitosan Gel in an in vivo Model of Cisplatin Ototoxicity
Source: Front Cell Neurosci. 2019 Jun 25;13:268. doi: 10.3389/fncel.2019.00268 (PMC6603134; doi:10.3389/fncel.2019.00268)
Supplement: Supplementary file 1 [file Table_1.docx]

Supplementary Material

# Supplementary Data

## Supplementary Tables

Supplementary Table S1. Quantification of hair cell loss in animals subjected to intratympanic (IT) administration of Thio-25. Percentage loss in the entire cochlea of outer hair cells in row 1 (OHC1), row 2 (OHC2), and row 3 (OHC3) and of inner hair cells (IHC), stratified by treatment (n=5).

|  | **Hair cell loss (%)** | | | | | | | | | | |
| --- | --- | --- | --- | --- | --- | --- | --- | --- | --- | --- | --- |
|  | **OHC1** | |  | **OHC2** | |  | **OHC3** | |  | **IHC** | |
| **IT** | **Mean** | **SD** |  | **Mean** | **SD** |  | **Mean** | **SD** |  | **Mean** | **SD** |
| None | 0.2 | 0.2 |  | 0.3 | 0.2 |  | 0.5 | 0.3 |  | 0.1 | 0.1 |
| Thio-25 | 0.4 | 0.2 |  | 0.3 | 0.3 |  | 0.7 | 0.5 |  | 0.0 | 0.1 |

Supplementary Table S2. Quantification of hair cell loss in animals subjected to intratympanic (IT) administration of Thio-40. Percentage loss in the entire cochlea of outer hair cells in row 1 (OHC1), row 2 (OHC2), and row 3 (OHC3) and of inner hair cells (IHC), stratified by treatment (n=5).

|  | **Hair cell loss (%)** | | | | | | | | | | |
| --- | --- | --- | --- | --- | --- | --- | --- | --- | --- | --- | --- |
|  | **OHC1** | |  | **OHC2** | |  | **OHC3** | |  | **IHC** | |
| **IT** | **Mean** | **SD** |  | **Mean** | **SD** |  | **Mean** | **SD** |  | **Mean** | **SD** |
| None | 0.6 | 0.3 |  | 0.6 | 0.3 |  | 0.6 | 0.1 |  | 0.1 | 0.1 |
| Thio-40 | 0.7 | 0.4 |  | 1.1 | 0.8 |  | 1.1 | 0.8 |  | 0.0 | 0.0 |

## Supplementary Figures

**
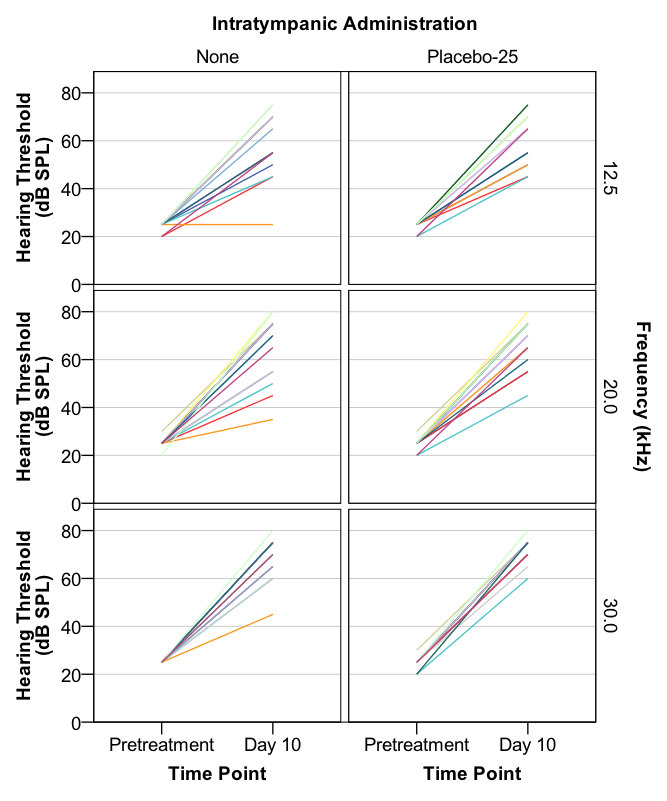
**

**Supplementary Figure S1.** Guinea pigs (n=15) were subjected to a single, unilateral intratympanic (IT) injection of Placebo-25, the vehicle of Thio-25, while the other ear was left untreated, serving as a control (None). The animals received a single high dose of cisplatin (8 mg/kg b.w., i.v.) 1 h later. Electrophysiological hearing thresholds (in decibel sound pressure level, dB SPL) assessed with air-conducted acoustically evoked auditory brainstem response (ABR) at 12.5, 20, and 30 kHz before and 10 days after IT administration are shown. Each color represents one animal. Fewer colors than 15 per graph are due to overlapping lines.

**
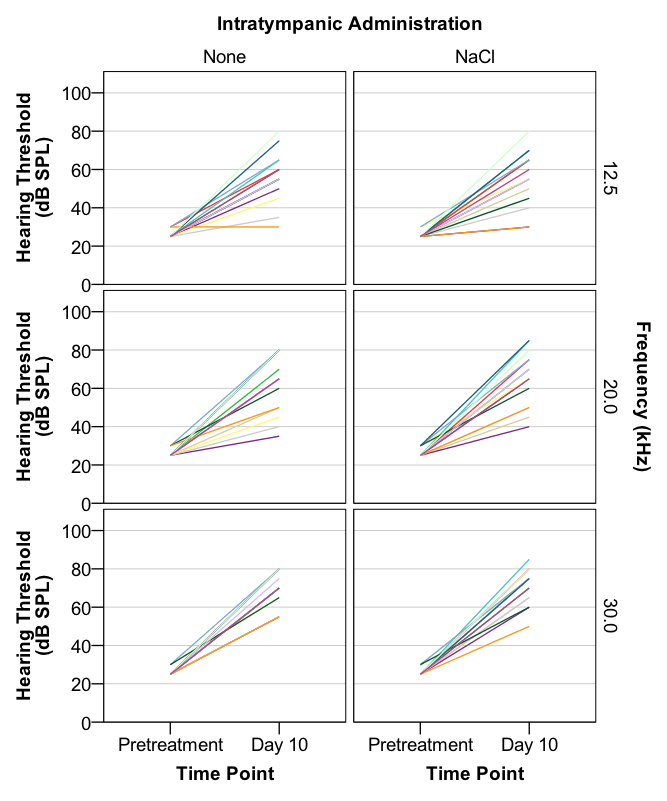
**

**Supplementary Figure S2.** Guinea pigs (n=15) were subjected to a single, unilateral intratympanic (IT) injection of aqueous sodium chloride (9 mg/ml; NaCl), while the other ear was left untreated, serving as a control (None). The animals received a single high dose of cisplatin (8 mg/kg b.w., i.v.) 1 h later. Electrophysiological hearing thresholds (in decibel sound pressure level, dB SPL) assessed with air-conducted acoustically evoked auditory brainstem response (ABR) at 12.5, 20, and 30 kHz before and 10 days after IT administration are shown. Each color represents one animal. Fewer colors than 15 per graph are due to overlapping lines.
